# Eating disorders and COVID-19 - different or just more?

**DOI:** 10.1007/s11845-024-03649-x

**Published:** 2024-03-16

**Authors:** Cathal Rafferty, Angela O’Donnell, Sally Campbell, Bohan Sun, Jenny King, Zeinab Ali, Diarmuid Lynch, Elizabeth Barrett, Sarah Richardson, Michelle Clifford, Fiona McNicholas

**Affiliations:** 1 School of Medicine, University College Dublin, Dublin, Ireland; 2 Department of Paediatric Liaison Psychiatry, Children’s Hospital Ireland, Crumlin Dublin 12, Dublin, Ireland; 3 Department of Paediatric Liaison Psychiatry, Children’s Hospital Ireland, Temple St. Dublin 2, Dublin, Ireland; 4 Lucena CAMHS, SJOG, Rathgar Dublin 6, Dublin, Ireland

**Keywords:** CAMHS, COVID-19, Eating disorders, Hospital admission, Referral rates

## Abstract

**Background:**

COVID-19 saw an increase in child mental health presentations internationally. Clinicians analogised the exponential increase in anorexia nervosa to a ‘tsunami’ or ‘outbreak’, raising parallel concerns regarding medical and psychological risks (Marsh in The Guardian, 2021; Leask in NZ Herald, 2021; Monteleone et al. in Eat Weight Disord 26(8):2443–2452, 2021) . It is unclear whether Ireland emulated this picture of increased referrals with increased medical compromise.

**Aims:**

This paper examines both rates and clinical profiles of child eating disorder presentations in the Republic of Ireland (ROI), across different clinical settings.

**Methods:**

Following ethical approval, retrospective chart reviews were conducted in a community eating disorder service and in two paediatric hospital settings. The time frame of the different studies ranged from January 2016 to December 2022.

**Results:**

Community eating disorder services saw significantly higher referral rates post COVID-19 (3.78/month vs. 2.31/month, *p* = 0.02), with a shorter duration of illness (4.8 months vs. 7.4 months, *p* = 0.001), but no significant difference in ideal body weight % (IBW%) at referral (85.32% vs. 83.7%, *p* = 0.1). Both paediatric hospitals witnessed significantly increased referrals post-COVID-19 (hospital 1; 4.38/month vs. 1.93/month, *p* = 0.0001; hospital 2; 2.8/month vs. 0.92/month, *p* < 0.0001), but no significant difference in IBW% at assessment (hospital 1; 82.7% vs. 81.39%, *p* = 0.673; hospital 2; 81.5% vs. 83%, *p* = 0.563). There was no significant difference in clinical profile, management, or duration of hospital stay.

**Conclusions:**

This study supports the growing consensus of a pandemic specific increase in eating disorder referrals to both medical and psychiatry services. However, there was little to indicate a change in clinical profile or severity. Ongoing monitoring of referrals is necessary to ensure adequate service availability and expertise.

## Introduction

In Ireland, evidence has emerged regarding the adverse impact of the COVID-19 pandemic and lockdown, on the mental health (MH) and wellbeing of children [4]. Referrals to Child and Adolescent Mental Health Services (CAMHS) subsequently increased, while anecdotal reports from clinicians reported an increase in referral complexity, citing a higher frequency of suicidal ideation and eating disorders [4]. Referrals to Irish paediatric emergency departments for suicidal ideation and self-harm also increased, with monthly attendance from June to December 2020 above that of 2019, at a time when presentations for all other medical reasons remained lower, suggesting a disproportionate effect of the pandemic on child MH [5]. Rates for eating disorders were also increased [5].

The HSE National Clinical Programme for Eating Disorders (NCPED) was established in 2014 and published its model of care in 2018 with the subsequent development of three specialist community eating disorder (ED) outpatient teams (two child and adolescent; one adult). This offered a unique opportunity to examine referrals to ED service pre and following COVID-19 onset and signaled an increased rate of referrals for all age groups [6]. Data from the hospital in-patient enquiry (HIPE) and the Health Research Board’s National Psychiatric In-patient Reporting System (NPIRS) provided further evidence for an increase in medical and psychiatric ED admissions for children, between 2019 and 2021, not evident among adult patients [7].

Driscoll and colleagues reported no difference in rates of medical or psychiatric co-morbidity but recognised as a limitation of their study an inability to examine important clinical parameters such as BMI, IBW%, or other aspects of medical instability [6]. Additionally, given the likelihood of increased referrals following service development, increased referrals rates are to be expected, and unless time series analysis is performed, any such increase cannot solely be attributed to COVID-19.

### Aim

This study addresses these previously omitted parameters by examining rates and clinical profile of children referred to a community eating disorder service and two paediatric settings in ROI.

## Methods

Study population included in this analysis (i) all children aged 5–17 referred to a CAMHS ED service between January 2018 and August 2021 [8], (ii) all paediatric ED referrals between January 2019 and December 2022 at CHI-Crumlin, and (iii) all referrals to CHI-Temple St between January 2016 and December 2020. Pre-COVID periods was defined as up to and including Feb 2020, with post COVID-19 from March 2020. This is in recognition of the fact that the first COVID-19 lockdown began in Ireland in March 2020. At the CAMHS ED service, it was recorded whether the patient stated that COVID-19 was a contributory factor to their illness. Ethical approval was granted by the respective research Ethics Committees.

## Data collection and statistical analysis

Retrospective chart reviews allowed data extraction onto a study proforma. Data was de-identified at source and transferred into an electronic data base for analysis using Statistical Package for Social Sciences (SPSS).

## Results

**Fig. 1:**
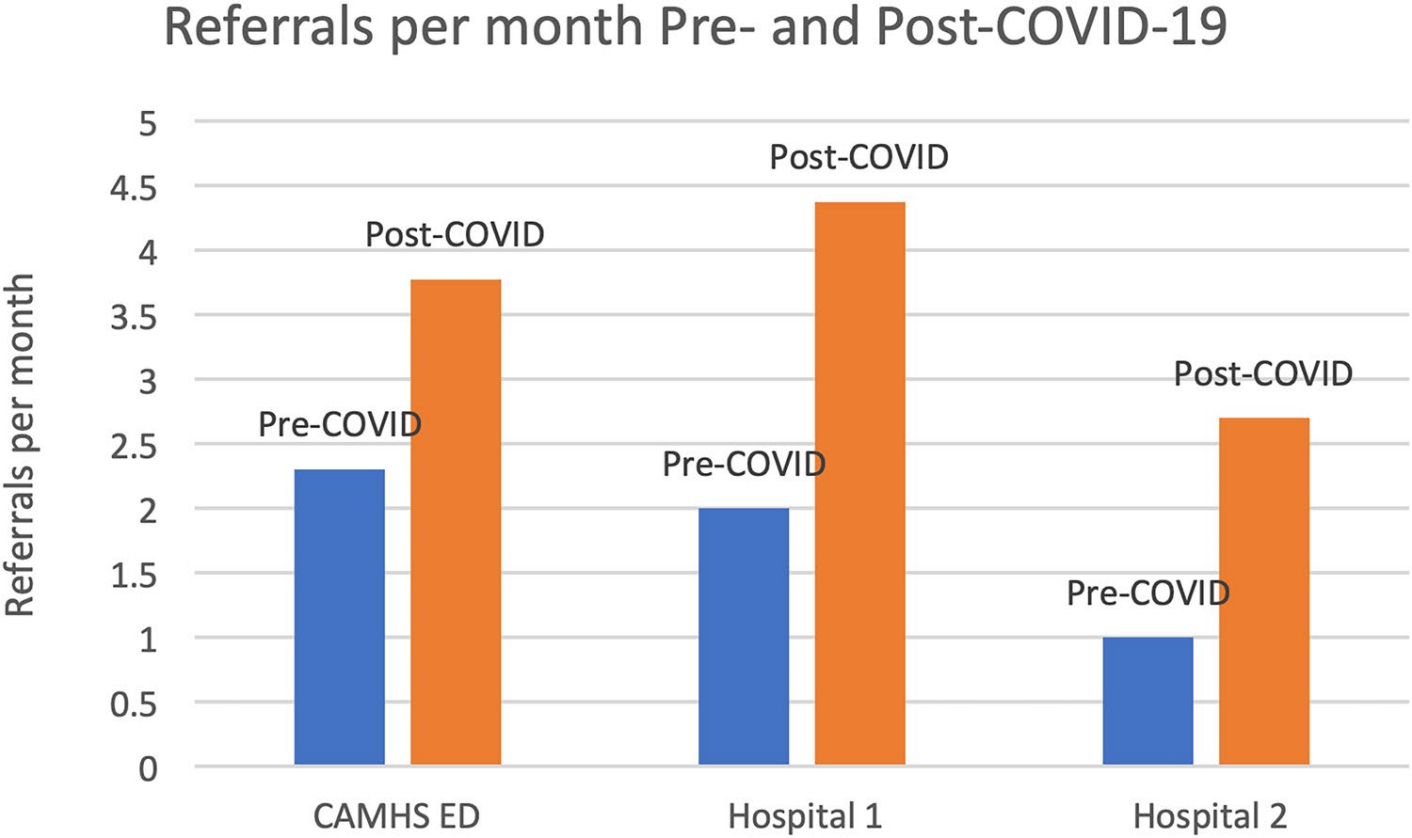
Referrals/month to CAMHS ED, Hospital 1 and 2 services pre and post COVID-19 onset

Clinical details for each of the services are provided below (Table 1, 2, and 3, Fig. 1). In each setting, most referrals were female (82.4–93.0%), with a mean age of 13.9–14.5 years and presenting with anorexia nervosa (61.2–93%). There was no significant difference over the time period studied in mean age, gender, ED type, and clinical management including length of stay, and NG feeding for hospital cases (Table 1, 2, and 3).

**Table 1:** Referral characteristics to CAMHS specialist ED service pre-COVID-19 and post-COVID-19

	Total sample (*N* = 128)	Pre-COVID-19 (*N* = 65)	Post-COVID-19 (*N* = 63)	Significance (chi-square or independent sample *t*-test)
Study time-period (months)	January 2018–August 2021 (44)	January 2018–February 2020 (26)	March 2020–August 2021 (18)	N/A
Referrals per month	2.9	2.31	3.78	0.021
Sex, *N* (%)	F: 119 (93.0)	F: 60 (92.3)	F: 59 (93.7)	Chi *x*^2^ (128, 1) = 0.088, *p* = 0.766, n.s.
Age year (mean ± SD)	14.5 (2.0) *N*: 128	14.6 (2.1)	14.4 (1.9)	*t*(128) = 0.551, *p* = 0.291, n.s.
Eating disorder type, *N* (%)	AN: 119 (93) ARFID: 5 (4) EDNOS: 4 (3)	AN: 62 (95.4) ARFID: 2 (3.1) EDNOS: 1 (1.5)	AN: 57 (90.5) ARFID: 3 (4.8) EDNOS: 3 (4.8)	Chi *x*^2^ (128, 2) = 3.518, *p* = 0.172, n.s.
Mental health comorbidity, *N* (%)	101 (78.9)	45 (77.6)	56 (80.0)	Chi *x*^2^ (128, 1) = 0.013, *p* = 0.908, n.s.
Psychotropic medication, *N* (%)	40 (31.3)	24 (41.4)	16 (22.9)	Chi *x*^2^ (128, 1) = 4.240, *p* = 0.039
Self-harm, *N* (%)	28 (21.9)	13 (22.4)	15 (21.4)	Chi *x*^2^ (128, 2) = 0.188, *p* = 0.910, n.s.
Prior admission for ED, *N* (%)	53 (41.4)	27 (46.6)	26 (37.1)	Chi *x*^2^ (128, 1) = 0.802, *p* = 0.370, n.s.
^Duration of weight loss pre−referral, months (mean±SD)^	^6.1 (4.0)^ ^*N*: 127^	^7.6 (4.6)^ ^*N*: 56^	^4.9 (3.0)^ ^*N*: 69^	^*t*(127) =3.758, *p* ≤0.001^
^Premorbid IBW% (mean±SD)^	^103.4 (14.2)^ ^*N*: 86^	^100.0 (15.8)^	^104.4 (13.6)^	^*t*(86) = −1.217, *p* =0.114, n.s.^
^Assessment IBW% (mean±SD)^	^85.3 (11.1)^ ^*N*: 128^	^83.3 (9.4)^	^87.0 (12.2)^	^t(128) = −1.904, *p* =0.030^

*IBW* ideal body weight, *AN* anorexia nervosa, *ARFID* avoidant restrictive food intake disorder, *OSFED* other specified feeding and eating disorder, *n.s.* non-significant, *SD* standard deviation

**Table 2:** CHI Hospital 1 cases pre-COVID-19 and post-COVID-19

	Total sample *N* = 177	Pre-COVID-19 *N* = 28	Post-COVID-19 *N* = 149	Significance (chi-square or independent sample *t*-test)
Study time period (months)	January 2019–December 2022 (48 m)	January 2019–February 2020 (14 m)	March 2020–December 2022 (34 m)	
Referrals/month	3.7	2.0	4.38	*p* = 0.0001 significant
Sex, *N* (%)	F:147 (83.1%)	F: 22 (78.6%)	F: 125 (83.9%)	Chi *x*^2^ (177, 1) = 0.171, *p* = 0.679, n.s.
Age, years (mean ± SD)	14.1 (1.74)	14.04 (1.67)	14.14 (1.76)	*t*(175) = − 0.302, *p* = 0.381, n.s.
Eating disorder type, *N* (%)	*N* = 177	AN: 20 (71.4%) BN: 0 (0%) ARFID: 2 (7.14%) OSFED: 4 (14.3%) Dx not stated: 2 (7%) *N* = 28	AN: 89 (59.7%) BN: 2 (1.34%) ARFID: 5 (3.36%) EDNOS: 40 (26.8%) Dx not stated: 13 (8.72%) *N* = 149	Chi *x*^2^ (3, *N* = 164) = 1.71, *p* = 0.633, n.s.
Length of stay	18.23 *N* = 175	12.61 days	19.28 days	*t*(175) = 1.339, *p* = 0.91 n.s.
Self-harm, *N* (%)	41 (23.2%) *N* = 177	4 (14.3%)	37 (24.8%)	Chi *x*^2^ (1, *N* = 177) = 0.949, *p* = 0.332, n.s.
Pre-morbid IBW% (± SD)	98.5% ± 13.6 *N* = 38	101.4% ± 9.3 *N* = 5	98% ± 14.2 *N* = 33	*t*(37) = 0.509, *p* = 0.307, n.s.
IBW% admission (± SD)	81.7% ± 10.8 *N* = 68	82.7% ± 11.0 *N* = 16	81.39% ± 10.8 *N* = 52	*t*(67) = 0.42, *p* = 0.337, n.s.
DC IBW (± SD)	86.2 ± 10.2 *N* = 62	86.9% ± 9.7 *N* = 14	86.0% ± 10.4 *N* = 48	*t*(61) = 0.282, *p* = 0.390, n.s.

*IBW* ideal body weight, *AN* anorexia nervosa, *ARFID* avoidant restrictive food intake disorder, *OSFED* other specified feeding and eating disorder, *n.s.* non-significant, *SD* standard deviation, *Dx* diagnosis

**Table 3:** CHI Hospital 2 cases pre-COVID-19 and post-COVID-19

	Total sample, *N* = 74	Pre-COVID-19 *N* = 47	Post-COVID-19 *N* = 27	Significance (chi-square or independent samples t-test)
Study time period (months)	January 2016–December 2020 (60 months)	Jan 2016–Feb 2020 50 m	March 2020–Dec 2020 10 m	
Sex, *N* (%)	*N* = 74 F: 61 (82.4%)	F: 40 (85.1%)	F: 21 (77.8%)	Chi *x*^2^ (1, *N* = 74) = 0.231, *p* = 0.631, n.s.
Age, years (mean ± SD)	13.9	13.9 ± 1.7 *N* = 47	13.8 ± 1.5 *N* = 27	*t*(72) = 0.003, *p* = 0 .499, n.s.
Referrals/month	1.23	1	2.7	
Eating disorder type, N (%)	AN = 61 (82.4%) ARFID = 6 (8.1%) OSFED = 7 (9.5%) *N* = 74	AN = 40 (85%) ARFID = 4 (9%) OSFED = 3 (6%) *N* = 47	AN = 21 (78%) ARFID = 2 (7%) OSFED = 4 (15%) *N* = 27	Chi *x*^2^ (2, *N* = 74) = 1.43, *p* = 0.480, n.s.
Proportion of referrals with AN, *N* (%)	61 (82.4%)	40 (85.1%)	21 (77.8%)	Chi x^2^ (1, N = 74) = 0.231, p = 0.631, n.s.
Length of stay (mean ± SD)	33.0 (22.2)	33.81 days (22.2)	31.81 (22.5)	*t*(72) = 0.369, *p* = 0.356 n.s.
IBW% admission (± SD)	82.5 (10.6)	82.6% ± 9.7 *N* = 45	82.2% ± 12.3 *N* = 25	*t* (69) = 0.180, *p* = 0.429, n.s.
DC IBW (± SD)	88.6 (9.7)	88.9% ± 9.6 *N* = 45	88.0% ± 10.2 *N* = 25	*t*(69) = 0.359, *p* = 0.361, n.s.
Any psychotropic medication	30 (40.5%)	21 (45%)	9 (33%)	Chi x^2^ (1, N = 74) = 0.506, p = 0.477, n.s.
NG	21 (28.4%)	14 (31%)	7 (26%)	Chi *x*^2^ (1, *N* = 72) = 0.040, *p* = 0.841, n.s.

*IBW* ideal body weight, *AN* anorexia nervosa, *ARFID* avoidant restrictive food intake disorder, *OSFED* other specified feeding and eating disorder, *n.s.* non-significant, *SD* standard deviation, *NG* nasogastric feeding

At the CAMHS specialist ED service (January 2018–August 2021), there was an average of 2.9 referrals per month during the study period. Patients were 93.0% females, with a mean age of 14.5 years. Then, 93% were diagnosed with anorexia nervosa and 78.9% had a mental health comorbidity.

At CHI Hospital 1 (Crumlin) (January 2019–December 2022), there was an average of 3.7 referrals per month. Then, 83.1% of patients were female, with a mean age of 14.1 years. Then, 61.6% of patients had a diagnosis of anorexia nervosa, and 23.2% of patients had self-harmed. The mean length of stay in hospital was 18.23 days.

At CHI Hospital 2 (Temple St) (January 2016–December 2020), there was an average of 1.23 referrals per month, and 82.4% of patients had a diagnosis of anorexia nervosa. There was a mean age of 13.9 years, and the mean length of stay in hospital was 33.0 days.

Cases referred to community services post-COVID-19 had a shorter duration of illness (mean 4.8 versus 7.4 months; *t* (91) = 2.151, *p* = 0.001) and lower rate of medication use (19% vs. 43%, *p* = 0.011). Many children (80%) self-declared COVID-19 as a contributory factor in the development of their ED.

At CHI Hospital 1 (CHI Crumlin), there was a significant increase in the number of referrals pre and post-COVID-19 (2.00/month versus 4.38/month, *p* = 0.0001).

## Discussion

Results from the 3 cohorts studied follow the typical pattern of what is already known about eating disorders clinically, with presentation during adolescence, a marked female predominance and presentation with low median body weights or %IBWs. Although there were a small proportion of cases with avoidant restrictive food intake disorder (ARFID), or other specific feeding and eating disorders (OSFED), most were for children with anorexia nervosa.

Consistent with national findings [6], referral rates significantly increased post-pandemic. Bodywhys, the ED association of Ireland, also reported an increased demand for their online support groups [9]. In line with the findings of Driscoll and colleagues in CAMHS setting, there was no difference in comorbid mental health difficulties. In our CAMHS cohort, available data suggested that significantly fewer children (19% vs. 43%, *p* = 0.011) were prescribed psychotropic medication post-COVID-19, perhaps linked to earlier recognition as reflected by a shorter duration of illness and faster referral rate.

School closures due to COVID were associated with huge lifestyle changes, parental home working, and closure of sporting facilities and opportunities [10]. The public health messaging at this time encouraged healthy eating and physical activity during lockdown. This is understandable given the high rates of obesity, and against a recognition that during the pandemic nearly half of American adults gained weight, especially those already overweight [11]. Weight gain was also linked to high rates of stress, depression, and anxiety [11]. However, potential adverse effects of weight stigma [12] were also noted and this calls for more careful public health messaging. Given the recognition that Ireland ranks as one of the highest levels for obesity in Europe, addressing this important health topic needs sensitive messaging to avoid increased disordered eating.

To what extent the increase in ED presentations post-COVID-19 is due to overzealous public health messaging in vulnerable groups, or is linked with a direct effect, is unknown. Although a bidirectional association between COVID-19 and psychiatric disorders, has been established, particularly linked to anxiety and mood disorders, eating disorders were not included [13]. A recent systematic review highlights several ways the pandemic may have indirectly adversely affected eating behaviours and disorders [14]. This included a preoccupation on weight/body shape, an over-reliance of dieting to manage weight following physical activity restrictions, reduced surveillance by primary care services linked to restriction or changes in healthcare provision, social isolation increasing stress and anxiety, and exacerbating symptoms in patients with EDs, and difficulty accessing certain food products. Qualitative studies are beginning to outline the myriad of lived experiences during COVID-19 for individuals and families who developed an ED or experienced a deterioration in a prior ED [15].

Findings from these 3 ROI cohorts mirror the international view, suggesting that COVID-19 has had a specific impact on referral rates of children with ED [6, 16, 17]. Some studies have further suggested an increase in illness severity, characterised by lower weights, increased medical instability, and increased requirement of hospitalisation [18, 19] as well as atypical case presentations [20]. Although our data reflect increased numbers of EDs, unlike previous suggestions, and in accordance with the NCPED data [6], our findings do not show any significant increase in medical compromise or complexity. Despite this, there was a shorter duration of weight loss prior to referral to the CAMHS specialist ED service. Further investigation of this may be warranted, and perhaps qualitative studies could assess whether clinicians became more alert to eating disorders and mental health difficulties among patients during the pandemic, leading to faster referrals to CAMHS services.

### Strengths and limitations of study

A strength of this study is the inclusion of children with eating-related difficulties across multiple clinical settings covering large catchment areas and the inclusion of clinical profiles. In addition, patient statements whether COVID-19 was a contributory factor to their illness were recorded at the CAMHS specialised ED service setting. The ED CAMHS service is a publicly funded service responsible for a catchment area of 260,560 children or 12.7% of all the children living in Ireland. Both paediatric hospitals are not confined to any catchment area. CHI Crumlin operates as an acute paediatric hospital with a 24-h emergency department as well as being the national centre for several paediatric specialties. CHI Temple Street located in Dublin inner city provides both quaternary and tertiary paediatric services. Limitations include a failure to capture children attending primary care or private services and admissions to specialist psychiatry inpatient units which were not captured in the 3 services examined. The time frame across each of the 3 settings is different, preventing combining all data. In addition, this study does not include data from the 3rd CHI hospital, CHI Tallaght.

## Conclusions

Longitudinal mental health surveillance data on the effects of the pandemic remains limited [21]. Given the recognition that the post-pandemic psychological effects often outlast and are more impactful than medical consequences, mental health morbidity is likely to rise [22]. Additionally, secondary mental health impacts associated with the public health response have also been noted [23]. Given Ireland was recognised to have one of the most prolonged and restrictive public health measures, further surveillance of the MH wellbeing, including referrals for EDs, is needed to ensure that these increased rates do not go unnoticed. This surveillance needs to be met with post-pandemic dedicated funding so that adequate services are in place to meet the needs of not only patients and families, but the clinical services responsible for their care.

## Data Availability

The authors can confirm that the data used for this study are available within the article and its supplementary materials.
